# Sexual and reproductive health: the science behind supplementation in aging

**DOI:** 10.1080/07853890.2021.1896092

**Published:** 2021-09-28

**Authors:** Alexandra Figueiredo, Ana I. Fernandes

**Affiliations:** PharmSci Lab, Centro de Investigação Interdisciplinar Egas Moniz (CiiEM), Egas Moniz Cooperativa de Ensino Superior, Caparica, Portugal

## Abstract

**Introduction:**

Throughout lifespan people are increasingly committed to take control of their own health and well-being, including fertility and sexual enhancement. Food supplements (FS) industry specifically caters for this segment market. However, FS are not medicines and thus, are not subject to the same strict regulations. Misbranded, unsafe and unlawful products are on the market [1], claiming scientifically unsubstantiated benefits, without disclosing potential side effects [2] and/or interactions [3], thus representing a public health hazard. The purpose of this work was to give an overview of the main FS intended for male and female sexual performance and reproductive health, available in Portugal.

**Materials and methods:**

Consumption data in pharmacies (2015–2018) was provided by ANF and inclusion criteria was FS which sold >1000 units/year. Formulas were analysed, to address the alleged bioactive substances, in a perspective of efficacy and safety.

**Results:**

Overall consumption of the FS studied has risen 48% in the years analysed and target mainly the male population (≈70% of products), although there are brands specifically designed for women. Botanicals, isolated or combined, used for sexual performance problems, such as erectile dysfunction and low sexual desire, mostly claim to: (a) improve genital blood flow [vasodilators as *Pausinystalia macroceras*, *Ferula asafoetida* L., *Pinus pinaster*, *Gingko biloba*, *Epimedium grandiflorum* L.], (b) increase testosterone levels [*Withania somnifera*, *Tribulus terrestris*, *Maca- Lepidium meyenii*], or (c) act as tonics/stimulants [*Eleutherococcus senticosus*, *Panax ginseng*, *Turnera diffusa*, guarana]. Plant extracts are at times associated with vitamins (A,D,E,C, folic acid), minerals (Zn, Se, Fe, Mg) and amino acids (arginine aspartate, L-carnitine). For fertility/climacterium associated symptoms, DHA, EPA, lycopene and CoQ10 are also found.

**Discussion and conclusions:**

Scientific evidence to support the claims of sexual enhancement is low/medium for all the plants, which however are not without harm. There is lack of studies in humans, no established daily doses and many of the side effects (e.g. severe hepatotoxicity of genus Pausinystalia; estrogen-like effect of Maca in gynaecological cancers) are not well documented. Associations of bioactives are common and increase the potential of side effects/toxicity and should be avoided. Relevant interactions with drugs [e.g. MAO inhibitors with Pausinystalia and P. ginseng; anticoagulants (e.g. warfarin) with *P. maritima*, *P. ginseng*, *E. senticosus*, *G. biloba*, *F. asafoetida*, *E. grandiflorum*; antihypertensive drugs with *T. terrestris*, *F. asafoetida*, *E. grandiflorum*, *W. somnifera* and *Pausinystalia*] are also possible, especially in case of polymedicated aging consumers. Moreover, since potentially harmful active pharmaceuticals, or its analogs, continue to be identified in FS, consumers should be aware of the risk in which they incur. Informed counselling by health-professionals is thus of the utmost importance and the subject should be taught to doctors, pharmacists, nutritionists and nurses.
